# Supplementary material for: Sustained RNA virome diversity in Antarctic penguins and their ticks
Source: ISME J. 2020 Apr 14;14(7):1768–82. doi: 10.1038/s41396-020-0643-1 (PMC7305176; doi:10.1038/s41396-020-0643-1)
Supplement: Supplementary file 1 — Supplemental Material [file 41396_2020_643_MOESM1_ESM.docx]

**Substantial and sustained RNA virome diversity in Antarctic penguins and their ticks**

Michelle Wille, Erin Harvey, Mang Shi, Daniel Gonzalez Acuña, Edward C. Holmes, Aeron C. Hurt

**Supplementary Material**

**Contents**

[Table S1. Alignment details for final phylogenetic trees presented in this manuscript. 2](#_Toc33189293)

[Table S2. Viral genomes described in this study 3](#_Toc33189294)

[Figure S1. Alpha diversity of avian viral families in Antarctic penguins 4](#_Toc33189295)

[Figure S2. Alpha diversity of avian viral genera in Antarctic penguins 5](#_Toc33189296)

[Figure S3. Non-metric multidimensional scaling (NMDS) plot applying the Bray Curtis dissimilarity matrix for viral abundance and diversity 6](#_Toc33189297)

[Figure S4. Alpha diversity of avian viral families in the tick *Ixodes uriae* 7](#_Toc33189298)

[Figure S5. Phylogenetic tree of the virus polyprotein of representatives of the vertebrate RNA virus family, the *Caliciviridae* 8](#_Toc33189299)

[Figure S6. Phylogeny of the virus polyprotein of selected members of the *Picornaviridae* 9](#_Toc33189300)

[Figure S7. Phylogeny of virus segment 2, containing the RdRp, of the *Picobirnaviridae* 10](#_Toc33189301)

[Figure S8. Phylogenetic trees of the of H5 and N5 influenza A viruses containing all wild bird sequences 11](#_Toc33189302)

[Figure S9. Phylogeny of the L gene (containing the RdRp) of avian avulaviruses 12](#_Toc33189303)

[Figure S10. Phylogenetic tree of the ORF1ab, containing the RdRp, of the *Coronaviridae* 13](#_Toc33189304)

[Figure S11. Phylogenetic tree of a short fragment of the ORF1b, containing the RdRp, of the Deltacoronaviruses 14](#_Toc33189305)

[Figure S12. Partial RdRp phylogeny of members of the avastroviruses 15](#_Toc33189306)

[Figure S13. RdRp phylogeny of viruses revealed in the tick libraries 16](#_Toc33189307)

Table S1. Alignment details for final phylogenetic trees presented in this manuscript.

| **Host** | **Group** | **Taxonomy** | **Nucleotide or amino acid** | **ORF** | **Alignment Length (including gaps)** | **Alignment length following trimAL** | **Fig #** |
| --- | --- | --- | --- | --- | --- | --- | --- |
| Penguin | ssRNA | Astroviridae, Avastroviruses | amino acid | ORF1ab (includes RdRp) | 2067 aa | 1507 aa | S12 |
| Penguin | ssRNA | Astroviridae, Avastroviruses | nucleotide | partial ORF1b (includes RdRp) | 403 bp |  | 6B |
| Penguin | ssRNA | Caliciviridae | amino acid | polyprotein (includes RdRp) | 3917 aa | 2374 aa | S5 |
| Penguin | ssRNA | Coronaviridae | amino acid | ORF1ab (includes RdRp) | 10805 aa | 6942 aa | S10 |
| Penguin | ssRNA | Coronaviridae, Deltacoronaviruses | nucleotide | partial ORF1b (includes RdRp) | 385 bp |  | S11 |
| Penguin | dsDNA | Alphaherpesviridae | amino acid | concatenation of glycoprotein B and major capsid | 2797 aa | 256 aa | 5B |
| Penguin | ssRNA | Orthomyxoviridae, Influenza A | nucleotide | HA | 1716 bp |  | S8A |
| Penguin | ssRNA | Orthomyxoviridae, Influenza A | nucleotide | NA | 1427bp |  | S8B |
| Penguin | ssRNA | Paramyxoviridae, Avulaviruses | nucleotide | F gene | 1626bp |  | 6A |
| Penguin | ssRNA | Paramyxoviridae, Avulaviruses | amino acid | L gene (includes RdRp) | 8017 aa | 6887 aa | S9 |
| Penguin | dsRNA | Picobirnaviridae | amino acid | Segment 2 (includes RdRp) | 768 aa | 533 aa | S7 |
| Penguin | ssRNA | Picornaviridae | amino acid | polyprotein (includes RdRp) | 6625 aa | 2347 aa | S6 |
| Penguin | dsRNA | Reoviridae, Rotavirus | amino acid | VP1 (includes RdRp) | 1198 aa | 1161 aa | 5A |
| Tick | dsRNA | Reoviridae | amino acid | VP1 (includes RdRp) | 3474 aa | 1042aa | 7A |
| Tick | ssRNA | Bunyavirales | amino acid | L protein (includes RdRp) | 3572 aa | 2507aa | 7B |
| Tick | ssRNA | Iflaviridae | amino acid | polyprotein (includes RdRp) | 5225 aa | 1045aa | S13 |
| Tick | ssRNA | *Phenuiviridae* | amino acid | RdRp | 1416 aa | 1133aa | S13 |
| Tick | ssRNA | *Rhabdoviridae* | amino acid | RdRp | 2296 aa | 1378aa | S13 |
| Tick | ssRNA | *Alphatetraviridae* | amino acid | RdRp | 7037 aa | 704aa | S13 |

# Table S2. Viral genomes described in this study

Please refer to the attached excel spreadsheet.


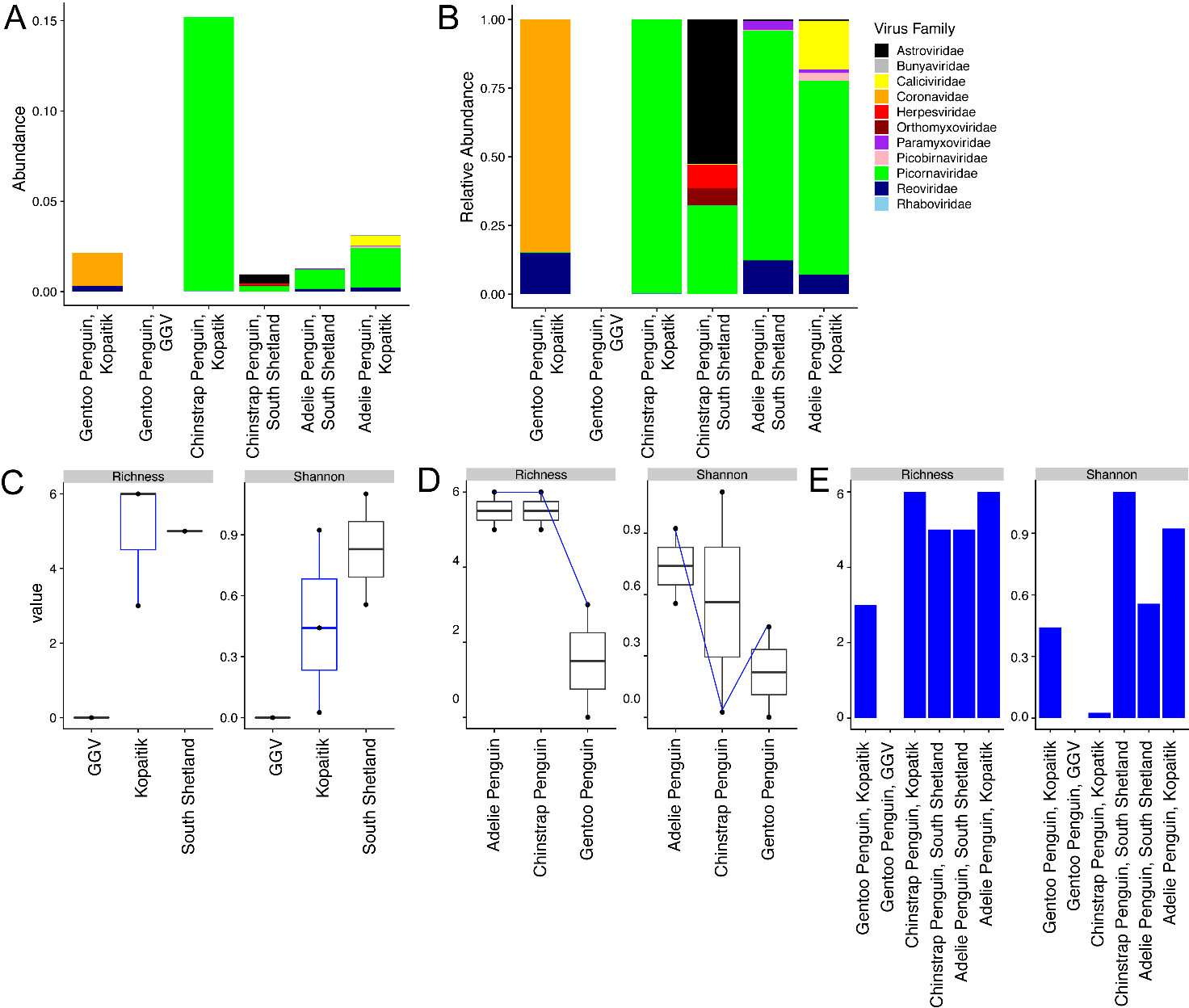


Figure S1. Alpha diversity of avian viral families in Antarctic penguins. Diversity and (A) abundance and (B) relative abundance of avian viral families. Alpha diversity metrics for (C) each location and (D) penguin species in this study Blue lines connect the alpha diversity of penguins sampled at Isla Kopaitik. (E) Alpha diversity metrics for each penguin library.


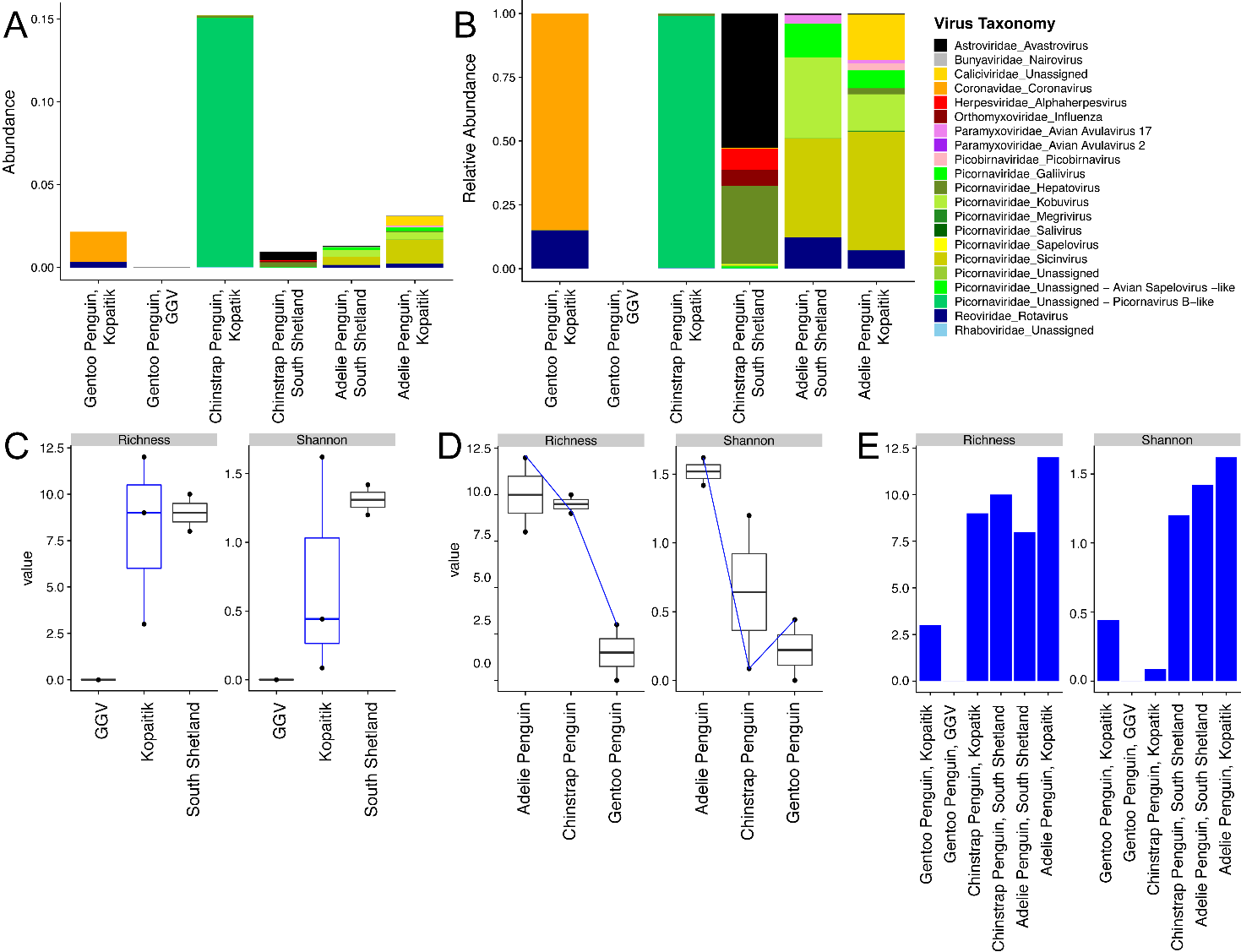


Figure S2. Alpha diversity of avian viral genera in Antarctic penguins. Diversity and (A) abundance and (B) relative abundance of avian viral genera. Alpha diversity metrics for (C) each location and (D) penguin species in this study. Blue lines connect the alpha diversity of penguins sampled at Isla Kopaitik. (E) Alpha diversity metrics for each penguin library.


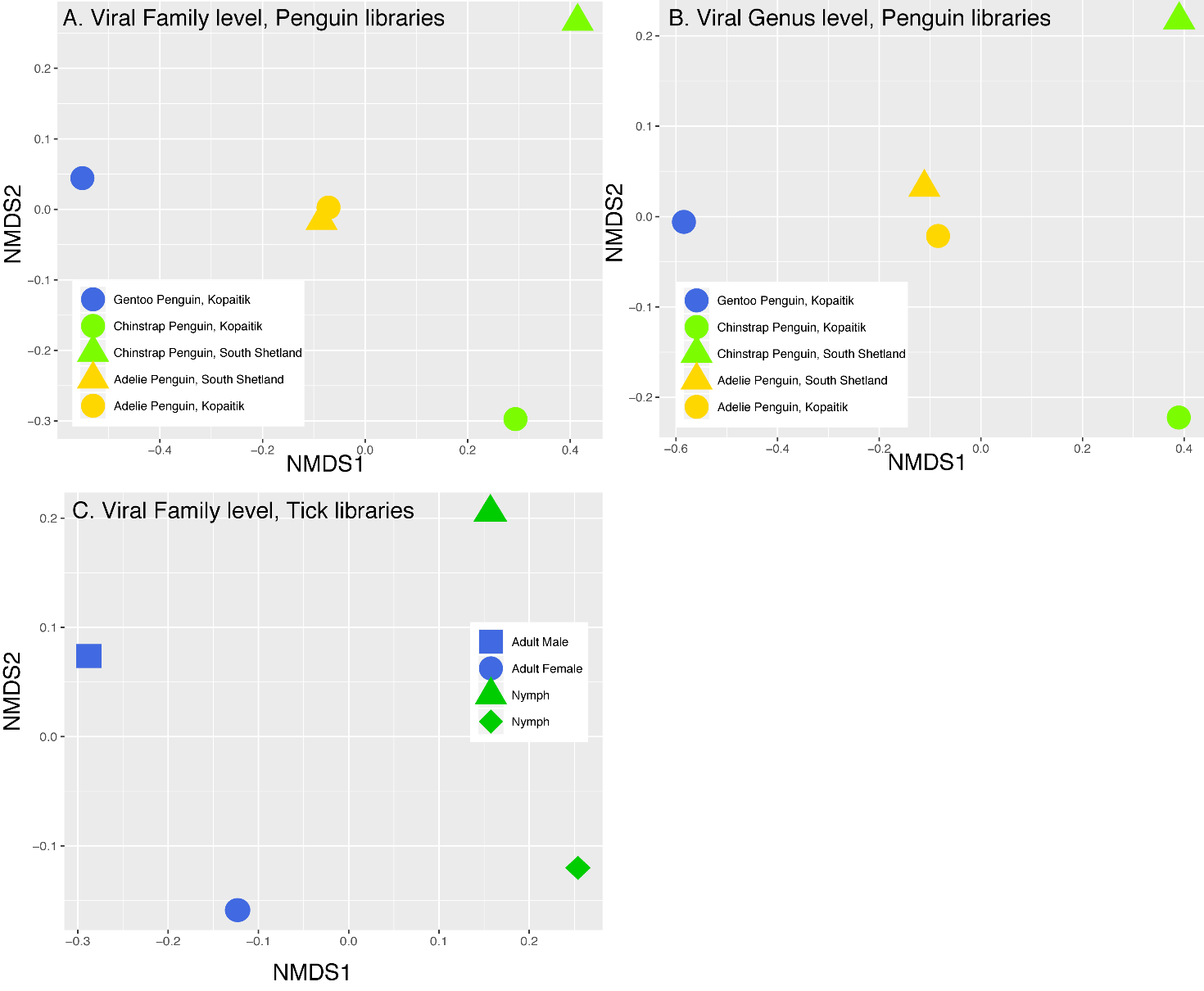


Figure S3. Non-metric multidimensional scaling (NMDS) plot applying the Bray Curtis dissimilarity matrix for viral abundance and diversity. (A, B) NMDS of penguin libraries in which (A) uses the viral family level and (B) uses viral genera level library composition. Gentoo penguins from GGV base are not included as they contained no avian viral reads. There is significant clustering of libraries when considering Host+Location at the viral family level (Adonis: Location R^2^=2.1923, p=0.03333; Host R^2^=0.601, p=0.050), but not when considering only Host or Location. There is no significant clustering at the level of virus genus. (C) NMDS of tick libraries considering the viral family level and there is not significant clustering.


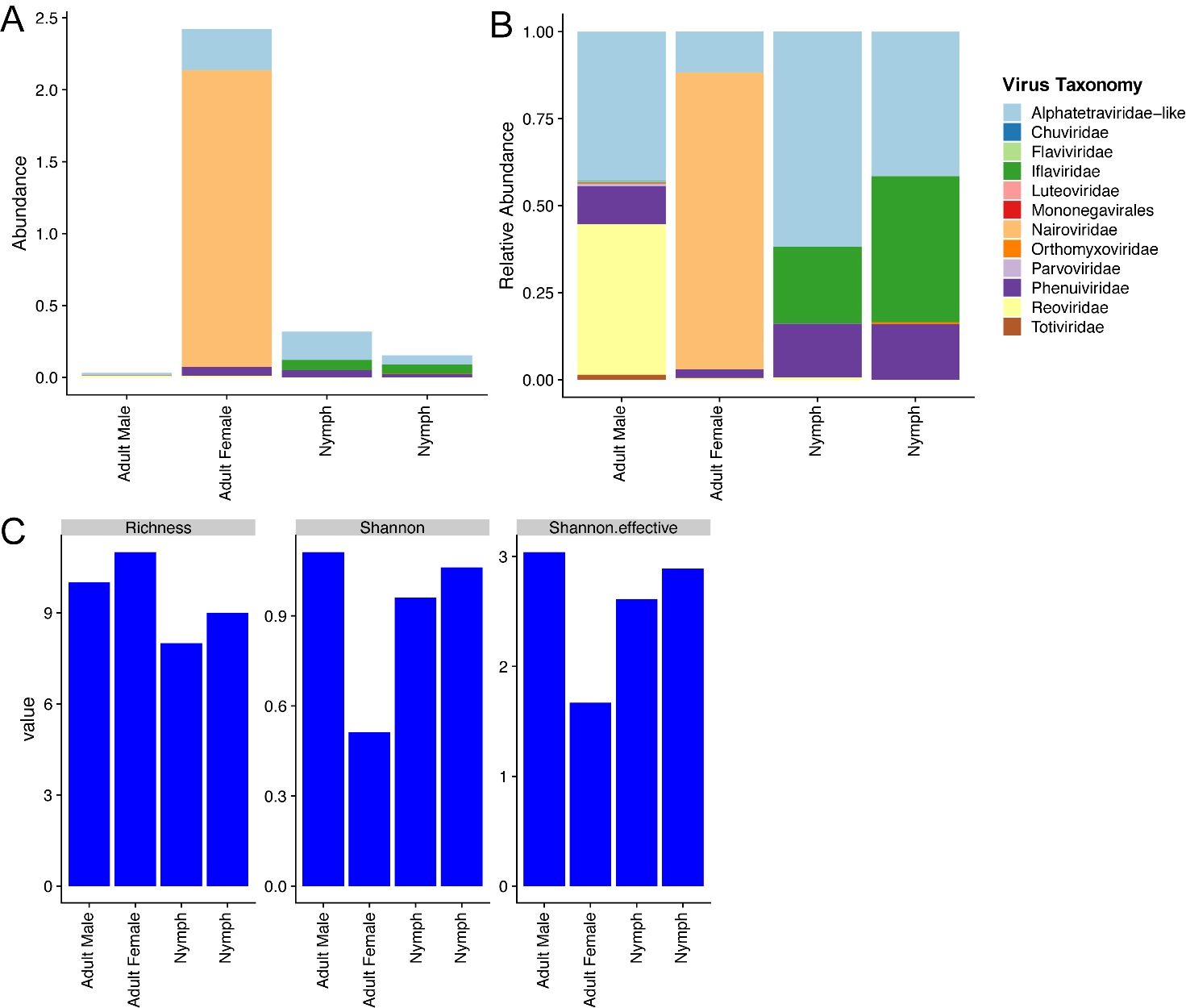


Figure S4. Alpha diversity of avian viral families in the tick *Ixodes uriae*. Diversity and (A) abundance and (B) relative abundance of avian viral families. (C) Alpha diversity metrics for each penguin library.


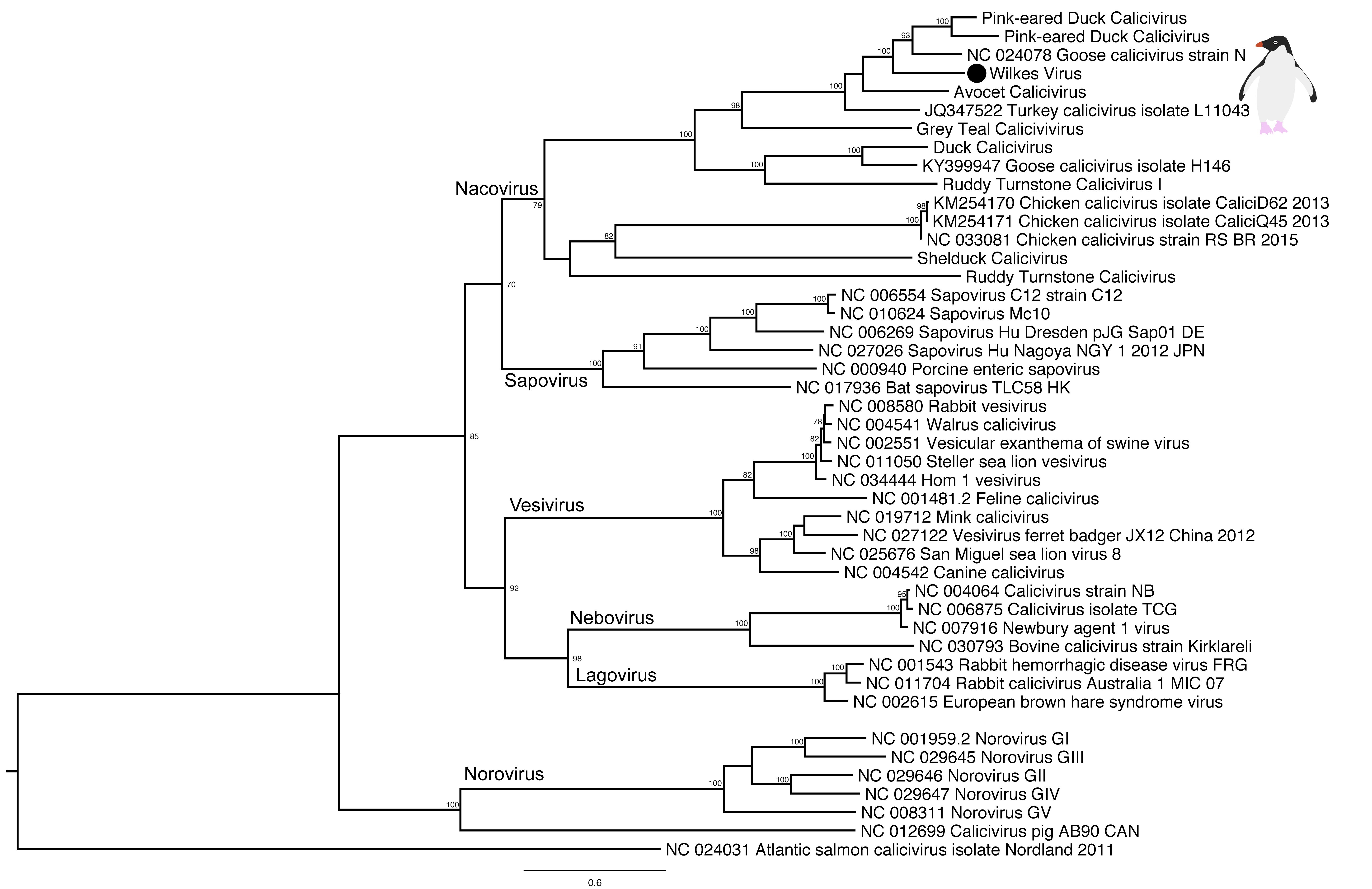


Figure S5. Phylogenetic tree of the virus polyprotein of representatives of the vertebrate RNA virus family, the *Caliciviridae*. Viruses identified in this study are denoted with a filled circle and in bold. The most divergent calicivirus, Atlantic Salmon calicivirus, was used as outgroup to root the tree. Bootstrap values >70% are shown for key nodes. The scale bar represents the number of amino acid substitutions per site


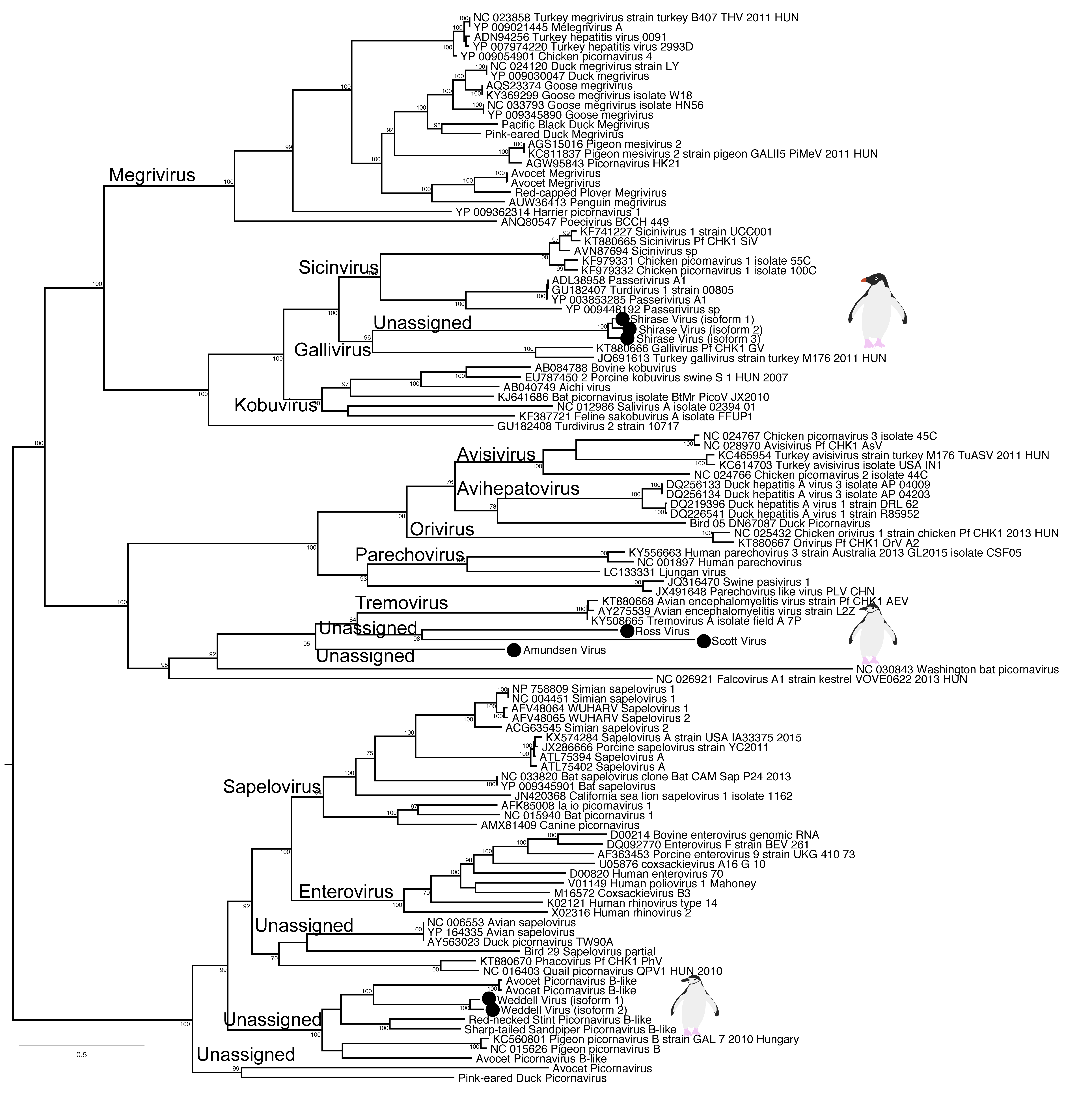


Figure S6. Phylogeny of the virus polyprotein of selected members of the *Picornaviridae*. The tree was midpoint rooted for clarity only. Viruses described in this study are adjacent to a filled circle. Bootstrap values >70% are shown for key nodes. The scale bar indicates the number of amino acid substitutions per site


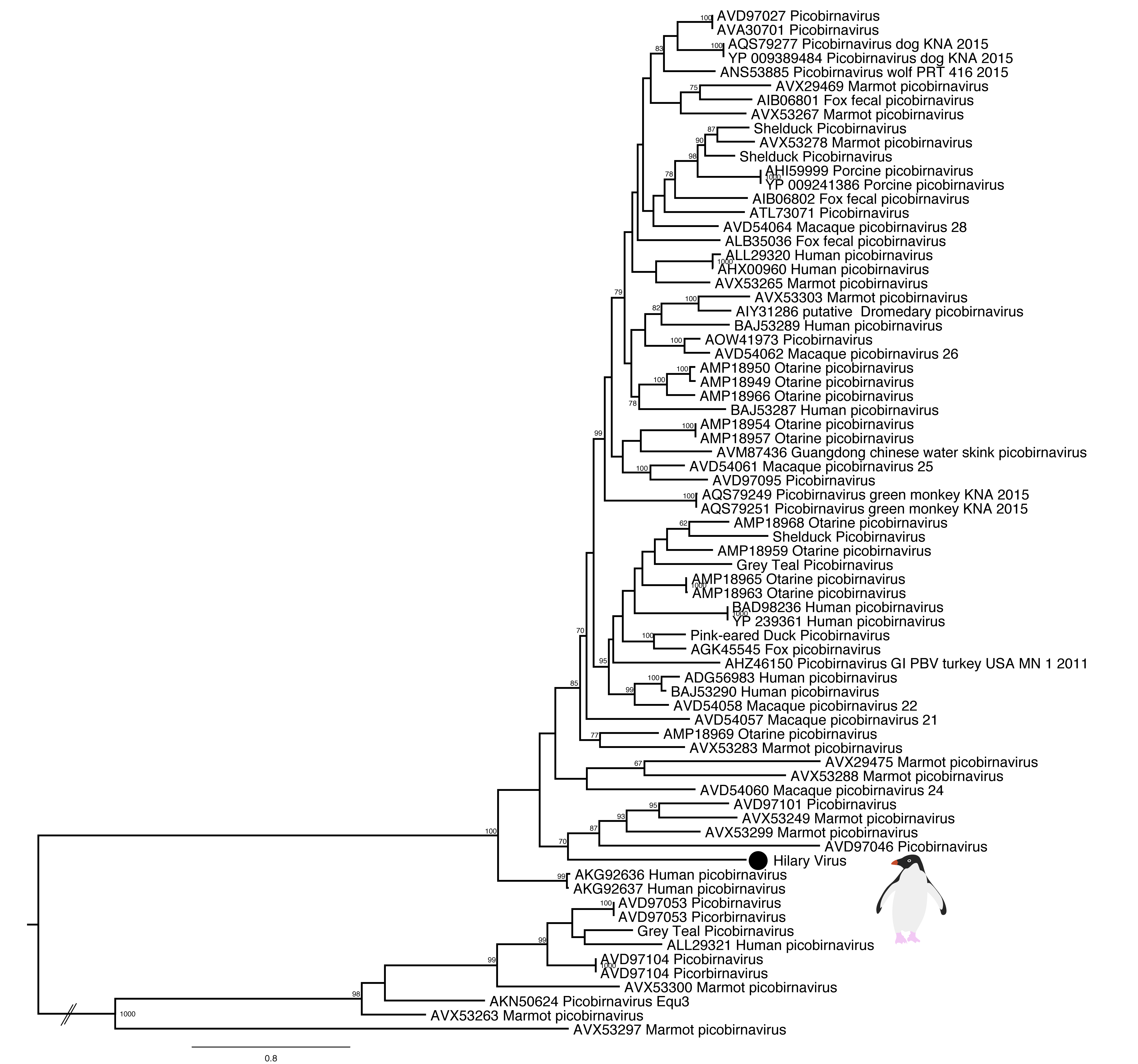


Figure S7. Phylogeny of virus segment 2, containing the RdRp, of the *Picobirnaviridae*. The tree was midpoint rooted for clarity only. Viruses described in this study are marked in bold, adjacent to a filled circle. Bootstrap values >70% are shown for key nodes. The scale bar indicates the number of amino acid substitutions per site.


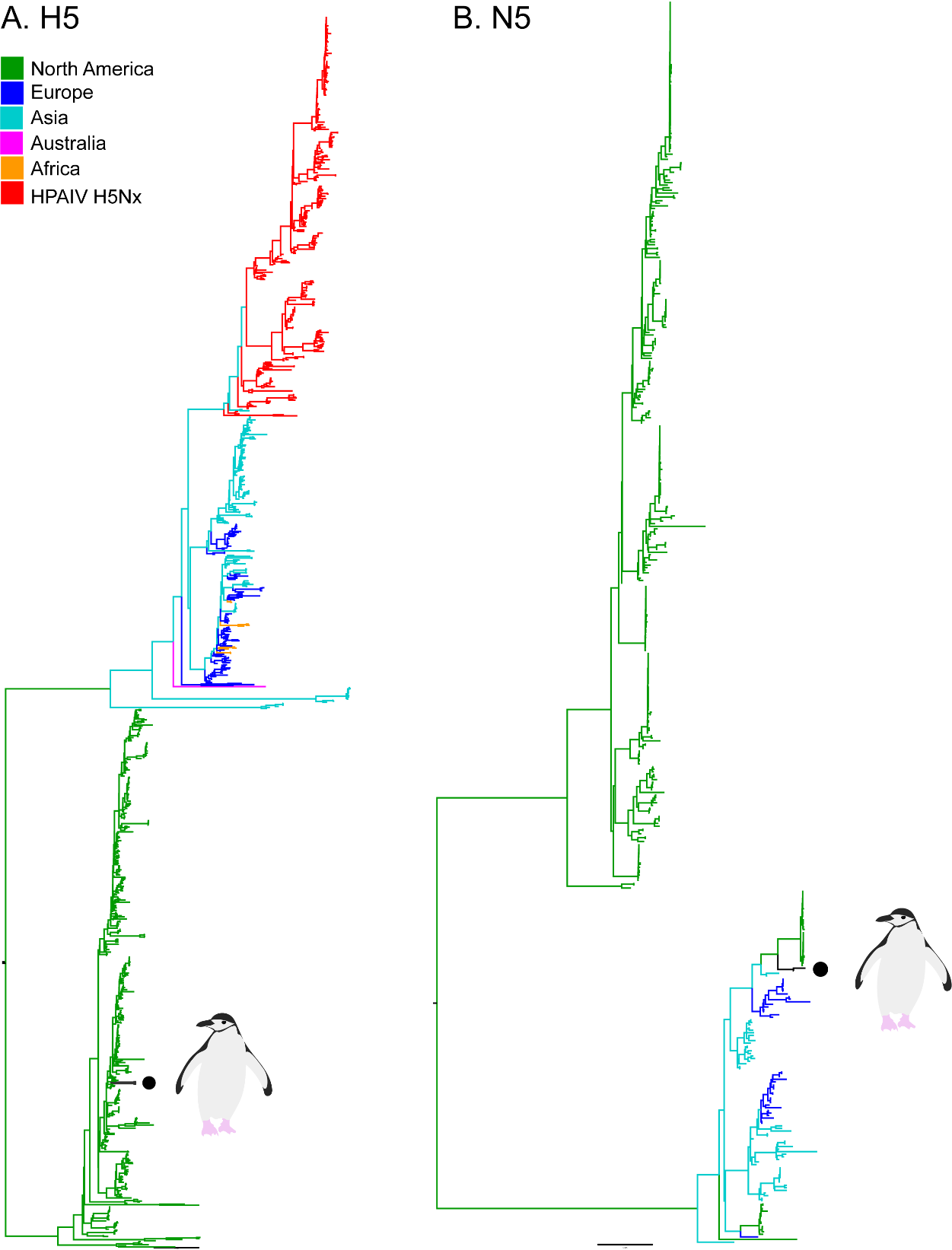


Figure S8. Phylogenetic trees of the of H5 and N5 influenza A viruses containing all wild bird sequences. The phylogenies were rooted on a major biogeographic division between Eurasia and the Americas. The phylogenetic position of A/Chinstrap Penguin/Antarctica/13/2015(H5N5) is denoted by a filled circle and is sister to a virus identified in Antarctic penguins in Hurt *et al*. (2016). The scale bare indicates the number of nucleotide substitutions per site.


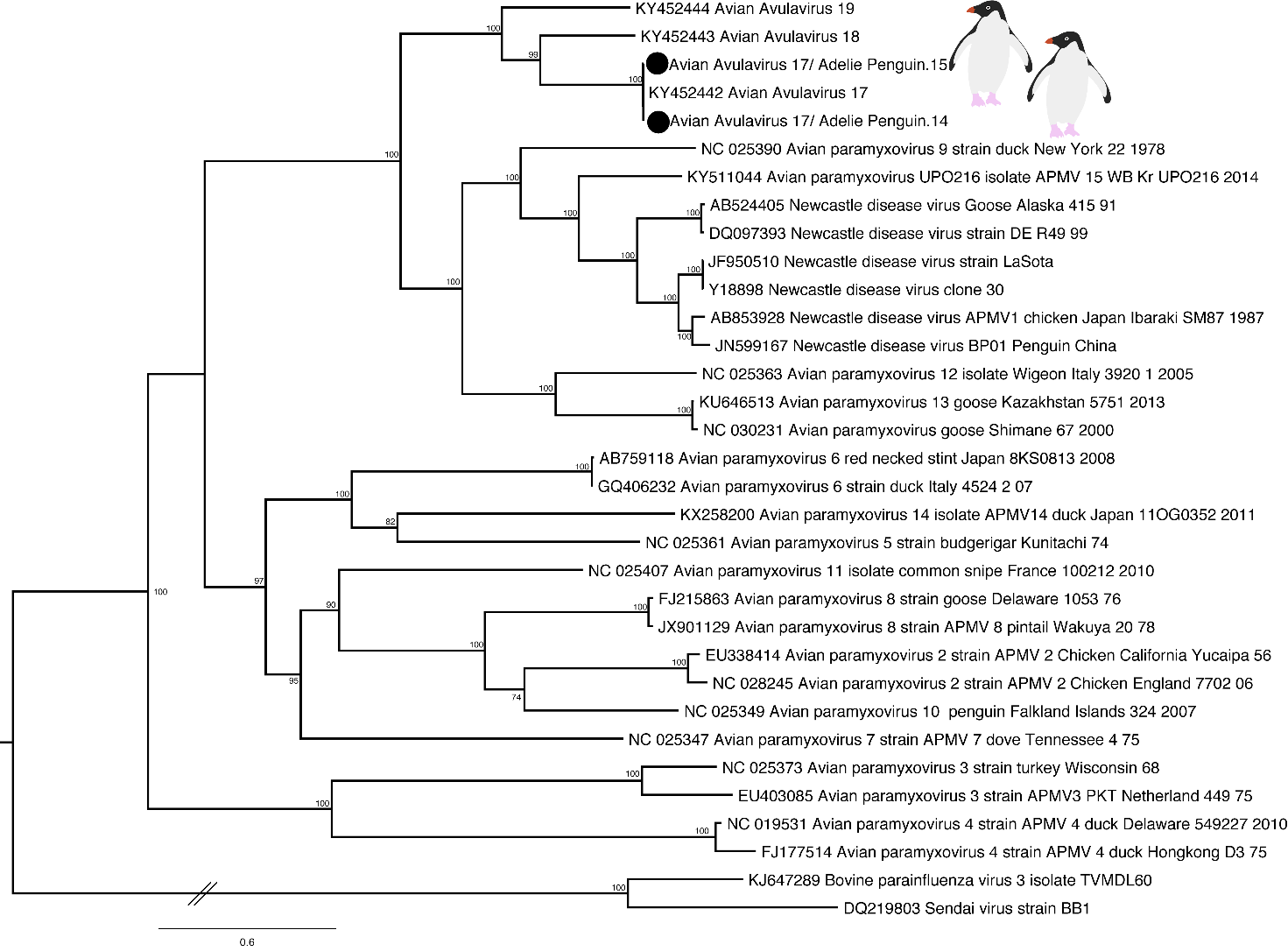


Figure S9. Phylogeny of the L gene (containing the RdRp) of avian avulaviruses. The tree is midpoint rooted for clarity. Viruses identified in this study are denoted with a filled circle and in bold. Bootstrap values >70% are shown for key nodes. The scale bar represents the number of amino acid substitutions per site.


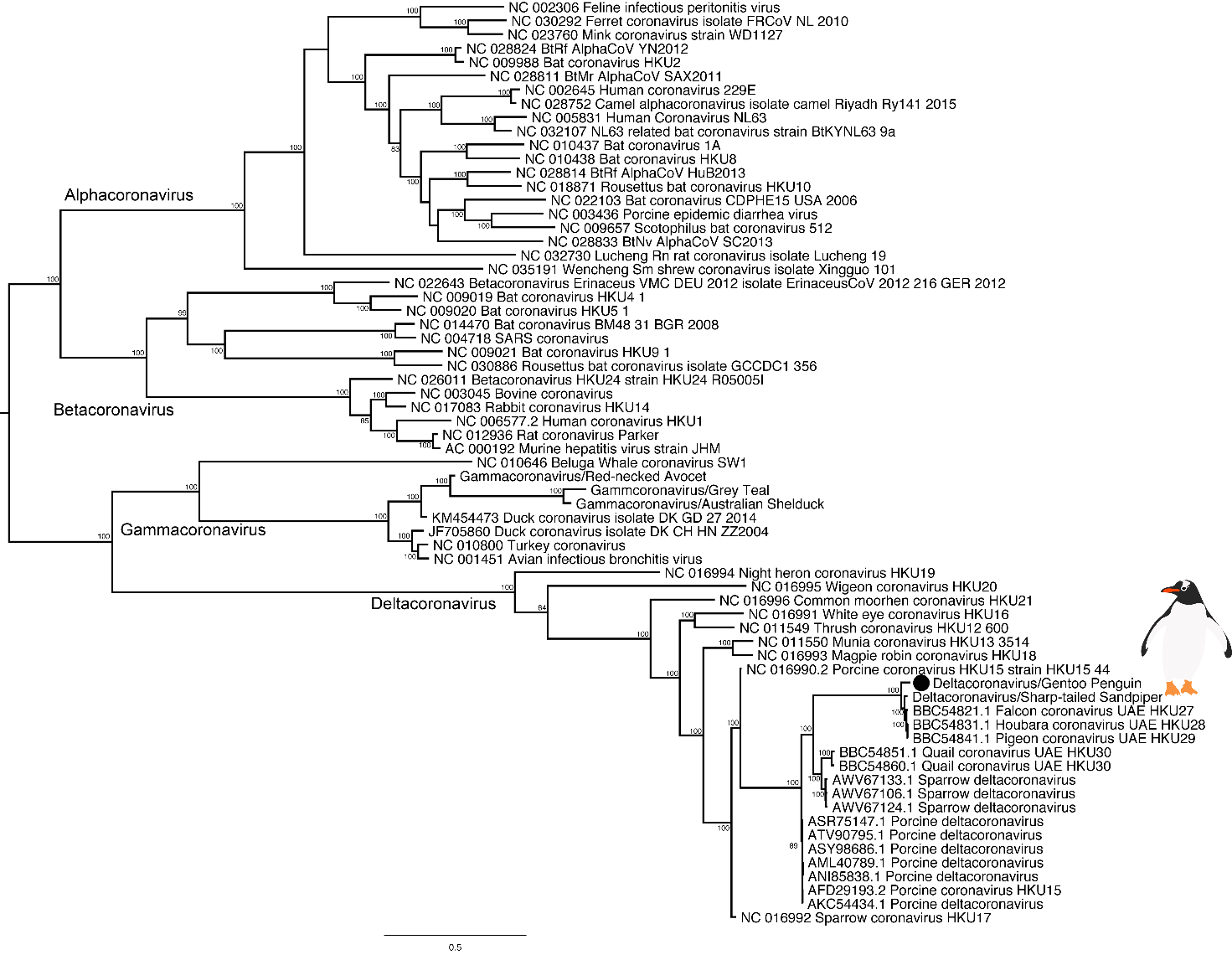


Figure S10. Phylogenetic tree of the ORF1ab, containing the RdRp, of the *Coronaviridae*. The sequences generated in this study are indicated by a filled circle and are in bold. The tree is midpoint rooted for clarity only. Bootstrap values >70% are shown for key nodes. The scale bar indicates the number of amino acid substitutions per site.


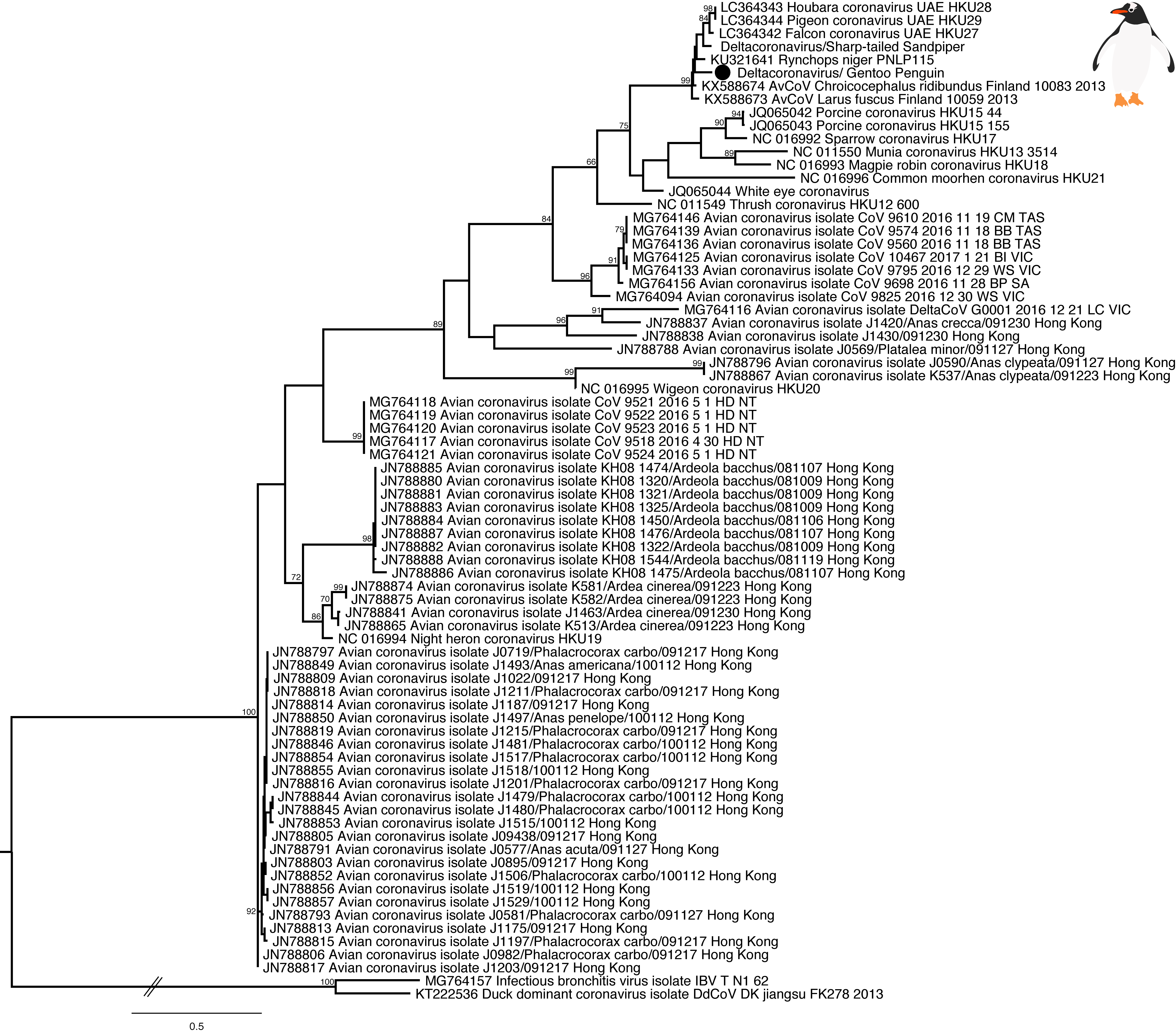


Figure S11. Phylogenetic tree of a short fragment of the ORF1b, containing the RdRp, of the Deltacoronaviruses. The sequences generated in this study are indicated by a filled circle and are in bold. Two gammacoronaviruses were set as the outgroup. Bootstrap values >70% are shown for key nodes. The scale bar indicates the number of nucleotide substitutions per site.


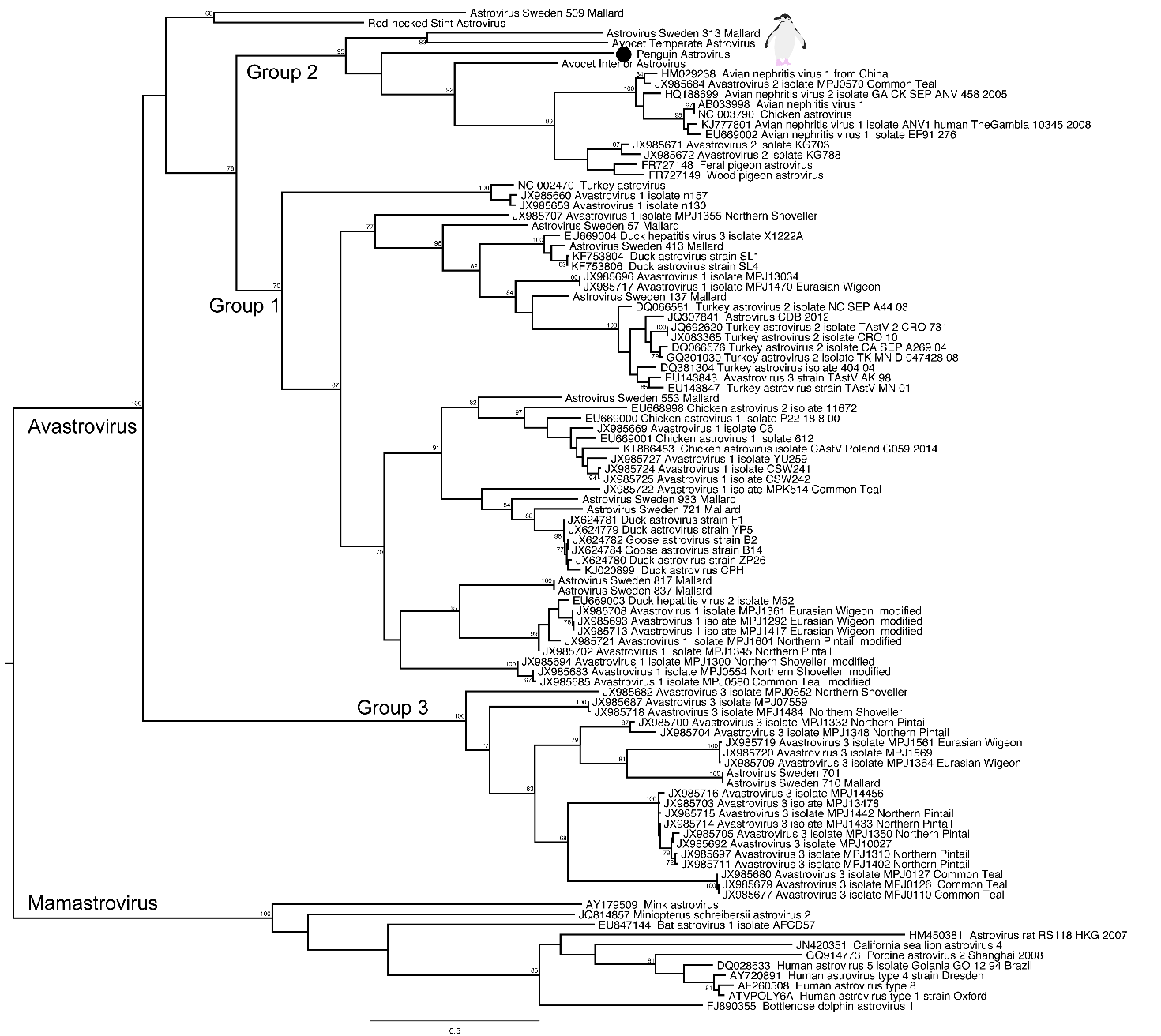


Figure S12. Partial RdRp phylogeny of members of the avastroviruses. The tree is rooted between the avian and mammalian astroviruses. The scale bar indicates the number of nucleotide substitutions per site. Viruses described in this study are marked in bold, adjacent to a filled circle. Bootstrap values >70% are shown for key nodes. The phylogeny of the full length polyprotein is presented in Figure 6B.


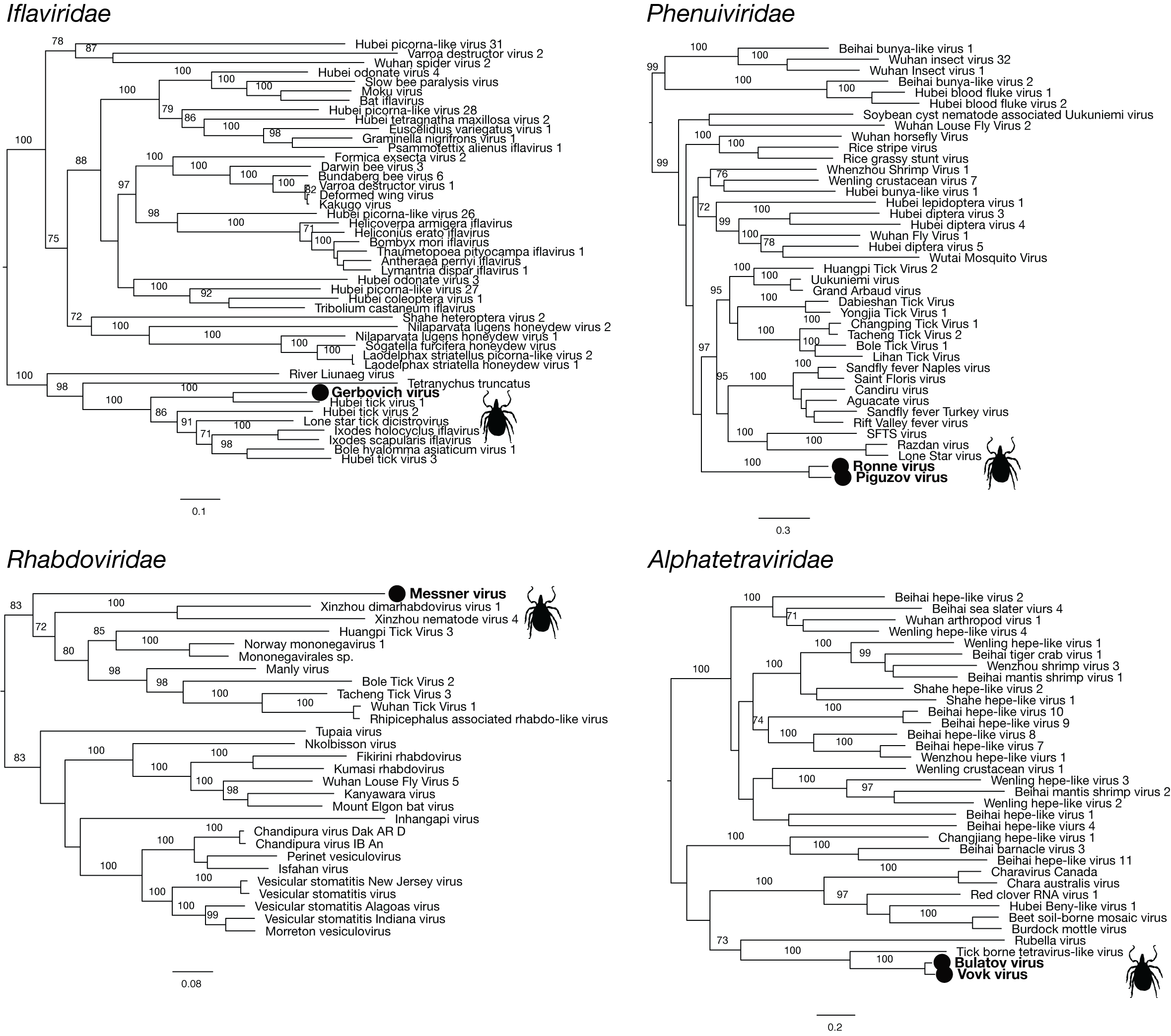


Figure S13. RdRp phylogeny of viruses revealed in the tick libraries. The trees are midpoint rooted for clarity. The scale bar indicates the number of amino acid substitutions per site. Tick viruses described in this study are in red. Bootstrap values >70% are shown for key nodes.
